# Adverse childhood experiences among females in substance use treatment and their children: A pilot study

**DOI:** 10.1016/j.pmedr.2021.101571

**Published:** 2021-09-28

**Authors:** Brittany T. Smith, Michael R. Brumage, Keith J. Zullig, Elizabeth A. Claydon, Megan L. Smith, Alfgeir L. Kristjansson

**Affiliations:** a Department of Social and Behavioral Sciences West Virginia University School of Public Health, 64 Medical Center Drive P.O. Box 9190, Morgantown, WV 26506-9190, United States; b Post-Deployment Health Services, Veterans Health Administration, Washington, DC, United States; c Department of Community and Environmental Health Boise State University Boise, ID, United States

**Keywords:** Adverse childhood experiences, Maternal substance use, Substance use treatment

## Abstract

Women with substance use disorder (SUD) often have experienced adverse childhood experiences (ACEs). The intergenerational nature of ACEs also put their children at risk for experiencing ACEs. However, no research has explored the prevalence of ACEs in children whose mothers have SUD. This study assessed ACE scores in mothers with SUD and their children and compared them with non-SUD participants. Females with SUD were recruited from a treatment center (n = 50) and compared to females without SUD from the same area (n = 50). The ACE scores of the participants and their children were measured as well as sociodemographic variables. ANOVA and Fisher’s Exact tests were used to examine univariate differences. Multivariate regression models assessed the difference in ACE scores between the groups and their children and the relationship between maternal and child ACE scores while including sociodemographic confounders. The mean ACE score was significantly higher in SUD participants (4.9, *SD* = 2.9) when compared to non-SUD participants (1.9, *SD* = 2.0) after controlling for sociodemographic variables (p < .01). Children of treatment participants also had significantly higher mean ACE scores (3.9, *SD* = 2.3) than children of comparison participants (1.3, *SD* = 2.0, p < .01). Maternal ACE score was positively related to children’s ACE score after controlling for sociodemographic variables. Given the intergenerational nature of ACEs and their high burden in both mothers and children in substance use treatment, these preliminary findings suggest that mother–child trauma-informed interventions may be appropriate for this population.

## 1 Introduction

Adverse childhood experiences (ACEs) are well-documented harmful events (Felitti et al., 1998, Hughes et al., 2017, Merrick et al., 2019, Nurius et al., 2019). ACEs are manifestations of childhood trauma that can be defined as an experience in which a child is exposed to emotional, physical, or sexual abuse, neglect, and/or household dysfunction before their 18th birthday (Felitti et al., 1998). Until recently, ACEs were thought to be rare occurrences, however recent estimates suggest that as many as 67% of the US population have encountered at least one of these events (Merrick et al., 2019, Merrick et al., 2018). Such experiences increase risks for many negative physical and mental health outcomes throughout the lifespan including, but not limited to, the five leading causes of death in the US (Jia and Lubetkin, 2020, Hughes et al., 2017, Merrick et al., 2019, Nurius et al., 2019). Research suggests individuals who experience multiple ACEs during childhood are at greater risk for developing various negative health outcomes in adulthood (Felitti et al., 1998, Jia and Lubetkin, 2020, Sonu et al., 2019, Merrick et al., 2017); including substance use disorder (SUD) (Stein et al., 2017, Quinn et al., 2019, LeTendre and Reed, 2017, Chandler et al., 2018, Bryant et al., 2020). The high prevalence of ACEs among the US general population and the negative outcomes with which they are associated constitutes a major, but largely unrecognized, public health problem, especially with regards to the ongoing substance use crisis.

The original study of ACEs by Felitti et al. (1998) found that those with 4 or more ACE points on a 0–10 scale were 4 to 12 times more likely to suffer from substance use problems in adulthood (Felitti et al., 1998). As of 2018, approximately 1 in 5 individuals in the US over the age of 12 had used illicit substances in the past year (Substance Abuse and Mental Health Service Administration, 2019). Subsequent studies have found that each ACE exposure notably increases the risk for substance use and the development of a substance use disorder (SUD) from adolescence through adulthood, even after controlling for key demographic variables (LeTendre and Reed, 2017, Dube et al., 2003). As an example, LeTendre and Reed found that for each one-point increase in ACE score the likelihood of developing a SUD in adulthood increased by 34%–47% (LeTendre and Reed, 2017).

With regards to gender differences, females have been found to be exposed to higher levels of ACEs and are more prone to develop ACE-related diseases throughout the lifespan when compared to males (Nakazawa, 2015). Substance use has also been defined as a coping mechanism more commonly used by females to cope with stress and feelings related to exposure to ACEs (Evans et al., 2017). Frequently experiencing certain ACEs, such as child abuse and neglect, has also been found to be associated with earlier initiation and escalation of substance use in a sample of females with SUD (Lotzin et al., 2019).

Children whose parents have a SUD have been found to be 2.7 to 4.2 times more likely to experience ACEs when compared to children with parents without SUD (Solis et al., 2012, Smith and Wilson, 2016). Studies have found that by early adulthood, twice as many children whose parents had an SUD had their own SUD when compared to the general population (Solis et al., 2012). Estimates suggest that approximately 70% of females receiving treatment for SUD have children (Niccols et al., 2012). Substance use among females often comes with distinct challenges for their children, since females often bear the majority of caregiver responsibilities (National Partnership for Women and Families, 2018). When under the influence of substances, mothers’ ability to care for their children is compromised, potentially damaging the mother–child bond and increasing the likelihood of ACEs in the offspring (Canfield et al., 2017). Mothers who have experienced ACEs, particularly those with SUD, have children who are at higher risk for experiencing ACEs (Murphy et al., 2014, Gannon et al., 2021, Schelbe and Geiger, 2017). This intergenerational cycle has been attributed to increased parenting stress and behavioral and emotional challenges experienced by mothers with ACEs (Murphy et al., 2014, Gannon et al., 2021). Despite this knowledge, limited research exists on the prevalence of ACEs in females with SUD and, to our knowledge, no studies have explored the ACE prevalence in their children.

The current study attempts to provide insight to address this gap. The purpose of this study was to: 1) describe the prevalence of ACEs among females with SUD and their children; 2) compare these rates to females without SUD and their children; 3) examine the relationship between maternal ACE score and children’s ACE score in both groups; and 4) assess the interaction between ACE scores among females with SUD compared to non-SUD participants and their children.

## 2 Methods

### 2.1 Study sample

Convenience sampling was employed to recruit two samples of women. This comparison study employed a sample of 50 female participants over the age of 18 that were recruited from a single inpatient abstinence-based peer recovery SUD treatment center in central West Virginia where 92 women were receiving treatment. A comparison group of 50 female participants without SUD who lived in the same area were also recruited from a Federally Qualified Health Center in the area. Data collection occurred from March 2019 – June 2019.

### 2.2 Procedures

Both SUD and comparison participants were recruited via institutional agreement between the West Virginia University (WVU) School of Public Health and the respective health care facilities. In both instances, participants were selected through reviews of their medical chart with the treating physician to ensure that study inclusionary criteria were satisfied. Informed consent was obtained by the research personnel. In the majority of instances, both treatment and comparison participants had children. All participants responded to the survey on an iPad using the Qualtrics internet-based platform. WVU Institutional Review Board approved all study protocols (IRB #1901428855).

### 2.3 Measures

ACEs were assessed using the Adverse Childhood Experience Questionnaire (Felitti et al., 1998). The measure queried participants in 10 areas regarding childhood abuse (sexual, physical and emotional), neglect (physical and emotional) and household dysfunction (incarcerated parent, parental substance abuse, parental mental illness, parental divorce or separation, and violence against mothers). Responses were scored with 1 = “yes” or 0 = “no”. Responses were summed to form a scale from 0 to 10 with higher scores indicative of greater ACE exposure.

The Center for Youth Wellness Adverse Childhood Experience Questionnaire (CYW ACE-Q) was utilized to obtain a composite ACE score for the children of study participants (Purewal et al., 2016). Study participants with children read 10 statements based on the original ACE measure and reported the total number of statements that applied to their child. The number reported was the composite ACE score for the child with higher scores also indicating greater ACE exposure.

The number of females who reported having children in the SUD group was 48/50 (96%) and 34/50 in the comparison group (68%). For mothers who had more than one child, the average of the children’s collective ACE score was used to indicate the score on the child’s ACE score for that particular participant.

### 2.4 Sociodemographic Control variables

Education, race, and age served as control variables in this study. Participants were asked to report the highest level of education they had completed. Possible responses included “never attended school or only attended kindergarten”, “grades 1 through 8 (elementary)”, grades 9–11 (some high school)”, “grade 12 or GED (high school graduate), “college 1 year to 3 years (some college or technical school)”, “college 4 years or more (college graduate)”, “some graduate or professional school”, and “graduate or professional degree (for example, MS, PhD, MD)”. This was recoded into a dichotomized variable where 0 = high school education or less and 1 = any higher education. Participants identified their race from the following responses, “White”, “Hispanic or Latino”, “Black or African American”, “Native American or American Indian” or “Other”. This variable was dichotomized and recoded as 0 = White and 1 = Other. Participants reported age in whole years.

### 2.5 Statistical analysis

Analyses began by assessing descriptive statistics for all study variables (Table 1), with frequencies for categorical variables and means and standard deviations for continuous variables. Fisher’s Exact test was used to assess group differences for categorical variables, and one-way analysis of variance to assess differences in continuous variables. Distributional properties were assessed for the dependent variables’ ACE scores and Child ACE scores with both ranging within limits on key measures (Skewness and Kurtosis < +/- 1.0). Four Ordinary Least Squares regression models were run to address study aims (see Table 2). Model 1 assesses the relationship between group (treatment = 1) and average ACE score after taking account of control variables; model 2 assesses the relationship between group (treatment = 1) and Child’s ACE score after taking account of control variables; model 3 adds participant ACE score to model 2. Finally, model 4 assesses the interaction relationship between group by ACE score on the Child’s ACE score. The interaction variable was computed using the mean-centered participant ACE score and group subtracted from 1.0. This approach brought the Variance Inflation Factor below the common threshold of 4.0 for all variables (Gujarati, 2009). All models were run in SPSS version 27 for Macintosh.

**Table 1:** Descriptive Statistics. All study variables.

**Variable**	**Range**	**Treatment group**		**Comparison group**		Sig.*
**Categorical variables**		**n**	**%**	**n**	**%**	
Education						
High school graduate or less	0–1	32	64.0	15	30.0	**
Any college		18	36.0	35	70.0	
Any children						
Yes	0–1	48	96.0	34	68.0	**
No		2	4.0	16	32.0	
Race						
White	0–1	42	84.0	49	98.0	*
Other		8	16.0	1	2.0	
IV Drug Use						
Yes	0–1	36	72.0	0	0.0	**
No		14	28.0	50	100.0	

**Continuous variables**	**Range**	**Mean**	**SD**	**Mean**	**SD**	
Age	18–64	33.2	8.40	38.0	13.33	*
ACE Score	0–10	4.9	2.91	1.9	2.00	**
Mean ACE Score of Child(ren)	0–10	3.9	2.35	1.3	2.04	**
Number of Children	0–8	1.9	1.08	1.6	1.57	Ns

*Ns = non-significant, * p < .05, ** p < .01.

**Table 2:** Results from OLS Regression analyses.

Variables	**Model 1: DV: ACEs score**		**Models 2**–**4. DV: Child ACEs score**					
	Unstand. B (SE)	P value	Unstand. B (SE)	P value	Unstand. B (SE)	P value	Unstand. B (SE)	P value
Education	−0.62 (0.53)	0.24	0.87 (0.57)	0.13	1.18 (0.52)	0.03	1.16 (0.53)	0.03
Race	−0.42 (0.89)	0.64	0.20 (0.98)	0.84	0.54 (0.90)	0.55	0.60 (0.91)	0.52
Age	−0.04 (02)	0.06	0.01 (0.03)	0.73	0.03 (0.03)	0.34	0.03 (0.03)	0.32
Group (treatment = 1)	2.64 (0.55)	<0.01	2.97 (0.64)	<0.01	1.81 (0.66)	0.01	1.92 (0.73)	0.01
ACEs score					0.38 (0.10)	<0.01	0.36 (0.12)	0.01
ACEs score * Group							0.10 (0.25)	0.70
*R*^2^	0.31		0.28		0.41		0.41	

## 3 Results

Table 1 includes descriptive statistics for all study variables and the results from the bivariate significance tests. As shown, treatment participants were significantly less likely to possess any college education compared to comparison participants, and they were significantly more likely to be non-white, slightly younger on average, and more likely to have children (p < .01). The mean ACE score of participants with SUD was significantly higher compared to comparison participants (4.9, *SD* = 2.9) vs. (1.9, *SD* = 2.0)) and so were their children’s ACE scores (3.9,*SD* = 2.3) vs. (1.3,*SD* = 2.0) (p < .01), respectively.

Multivariate models 1 and 2 (Table 2) affirmed this difference after accounting for the sociodemographic control variables. The SUD group, on average, scored 2.64 points higher on the 0–10 point ACEs scale after accounting for control variables. This difference was 2.97 points for the children’s ACE scores. Model 3 shows the relationship between participant ACE score and Child ACE score (β = 0.38,p < .01), suggesting that for each increase of 1 in mother’s ACE score, their children’s ACE score increased by 0.38 after taking account of other variables in the model. Model 4 tested if the relationship between ACE score and Child ACE score differed by group. As shown, the main effects from both ACE score and group remain significantly related to Child ACE score but the interaction variable for ACE score by group was non-significant (p = .70). Model 1 explained 31% of the variance, while Model 4 explained 41%.

## 4 Discussion

This study sought to describe the prevalence of ACEs in females in treatment for SUD and their children, and then to compare this prevalence to comparison participants. In addition, the study also sought to assess the relationship between maternal ACE scores and child ACE scores. Whereas two previous studies have explored ACEs scores among women in treatment for SUD (Gannon et al., 2021, Winstanley et al., 2020); no research has explored ACEs scores among their children. ACE prevalence was significantly higher among females in treatment for SUD compared to comparison participants as were their children’s ACE scores compared to the children of comparison participants. A positive relationship between maternal ACE score and children’s ACE score was also found. In the final model (Model 4), the interaction between ACE score and group was not significant further emphasizing that mothers with higher ACE scores are more likely to have children with higher ACE scores while mothers with lower ACE scores are more likely to have children with lower ACE scores.

The findings from this study are consistent with previous research that explored ACE prevalence in females in SUD treatment (Gannon et al., 2021, Winstanley et al., 2020). For example, Winstanley (Winstanley et al., 2020) found a mean ACE score of 4.5 in a rural sample of women (Winstanley et al., 2020) and Gannon and colleagues (Gannon et al., 2021) found a mean ACE score of 4.3 in a more urban sample, while also showing that their sample’s ACE burden was higher than in the general population (Gannon et al., 2021). Observing such a profound ACE prevalence in this population raises alarm for both parenting and substance use outcomes. Evidence has shown that poor substance use treatment outcomes, such as relapse, challenges with comorbid mental illness, and lower levels of improvement in treatment, are associated with high ACE prevalence (Sacks et al., 2008, Derefinko et al., 2019, Rosen et al., 2002, Farrugia et al., 2011, Kumar et al., 2016). The literature on trauma and parenting has consistently found that high levels of ACE are also linked to increased parenting stress, insecure parent–child attachment, negative parenting behaviors, and fear and confusion surrounding discipline (Slesnick and Zhang, 2016, Herbell and Bloom, 2020, Moe et al., 2018). Like many other consequences of experiencing numerous ACEs, these outcomes could be attributed to the dysregulation and hyperactivity of the stress response that is developed when significant adversity occurs (Lê-Scherban et al., 2018). This dysregulation has been found to result in changes in brain architecture and chemistry, such as the inability to produce certain neurotransmitters (Maté, 2008). This may result in seeking external supplements, such as substances, to compensate for the brain's altered functioning (Maté, 2008). This is often overlooked and untreated, and therefore may explain why many females with high ACE prevalence report using substances to self-medicate (Evans et al., 2017, Garland et al., 2013). Given that substance use is an added stressor with potentially negative consequences for parenting, this study’s results lend support to the idea that this may lead to cycles of further substance use and intergenerational trauma that can create challenges for mothers in treatment and their children.

Children with parents who have SUD are known to be at higher risk for ACEs (DeLisi et al., 2019, Dube et al., 2001, Anda et al., 2002).

This study underlined this risk by being the first study to explore ACE prevalence in this population. Results suggest that children of females in SUD treatment have concerning levels of ACE exposure, or a 3.9 score on average. Conversely, children of mothers without SUD from the same area as the SUD study participants had ACE scores three times lower than children of non-SUD participants. This is concerning as ACE research has demonstrated that those with ACE scores that are 4 or greater are at the highest risk for negative outcomes (Felitti et al., 1998, Dube et al., 2003). The likelihood of SUD, attention-deficit/hyperactivity disorder, depression, anxiety, behavioral disorders, and school drop-out has been well documented in children who have a high ACE scores (Uddin et al., 2020, Shin et al., 2018, Petruccelli et al., 2019). Parenting stress, which is more prominent in parents who use substances, has been found to play a mediating role in many of these relationships (Uddin et al., 2020).

.Both ACEs and substance use have been identified as public health problems, which can affect multiple generations (Knight et al., 2014, Meulewaeter et al., 2019). In the current study, maternal ACE score was strongly related to child’s ACE score, whether in treatment for SUD or not, supporting the notion that ACEs have an intergenerational component. Other studies provide similar results that demonstrate parents with high ACE prevalence are more likely to have children who experience ACEs (Schelbe and Geiger, 2017, Lê-Scherban et al., 2018, Schofield et al., 2018). One study that explored ACE scores across three generations found that later generations experienced more ACEs than the first generation (Grest et al., 2021). Our findings support the notion that intergenerational continuity of ACEs could contribute to the intergenerational occurrence of SUD since the link between SUD and ACEs is well established (LeTendre and Reed, 2017, Dube et al., 2003). Mood and anxiety disorders have been found to be pronounced meditators in the temporal relationship between ACEs and SUD (Douglas et al., 2010). Therefore, early therapeutic interventions that promote resilience and post-traumatic growth in children with mothers in SUD treatment could be a promising way of preventing or significantly reducing mood disorders, anxiety disorders and SUD in the next generation.

These findings add new knowledge to ACE science by providing preliminary prevalence data for children with mothers in SUD treatment. While previous research has found elevated risk for ACEs in this population of children, our study is the first to explore their ACE prevalence. Our study also expands the ACE literature by demonstrating that maternal ACE score is positively associated with children’s ACE score in the female SUD population.

The novel findings of this study suggest that trauma-informed interventions that directly target both the mother and child could be beneficial to this exclusive population with high ACE burden. Mothers have been found to have better substance use outcomes, such as faster declines in substance use, when family-based therapy including children was a part of the treatment program (Slesnick and Zhang, 2016). Parenting interventions targeting mothers in substance use treatment have also shown positive results. *Mothering from Inside Out* and *Mindfulness Based Parenting* are both interventions for mothers in substance use treatment which aim to enhance parenting skills and promote more securely attached relationships between mother–child dyads. Studies found that these interventions promoted healthier relationships between mothers and children (Suchman et al., 2018, Suchman et al., 2017, Gannon et al., 2019). While showing a positive impact on the mother–child relationship, most parenting interventions do not directly engage children. In qualitative studies, children of parents with SUD have reported numerous difficulties such as lack of support services, increased mental illnesses, SUD, parentification, parenting-related fears and confusion surrounding their relationships with their parent with SUD (Tedgård et al., 2019, Tedgård et al., 2018, Wangensteen et al., 2019). *The Children Program* implemented by the Hazelden Betty Ford Foundation is a two to three-day program for children with parents who have SUD (Arria and Mericle, 2014). An evaluation of this educational program found that behavioral and emotional problems were reduced significantly and that family functioning increased (Arria and Mericle, 2014).

The types of interventions mentioned above are rare and either target the mother or the child but not both, with children having far fewer resources (Daley et al., 2018). Our results suggest that providing interventions for the mother, child, and the mother child-dyad should receive greater attention as a potential way to prevent and treat SUD in families. Future research should work to develop and evaluate SUD treatments that treat the trauma experienced by females with SUD and their children.

## 5 Limitations

This study is not without limitations. First, due to our cross-sectional study design, causation cannot be determined from these results although it appears plausible that the transmission of ACE moves from mothers to children rather than vice versa. Second, the generalizability of the results is limited because of the small, predominately white, rural sample. Third, participants were asked to self-report on past experiences increasing the likelihood of recall bias. Fourth, the age of the children was not recorded, which limits our ability to report and compare potential developmental differences. Fifth, the matching of SUD participants and the comparison participants was not done on a one-to-one basis, which would be preferable in future studies. Lastly, child ACE score was reported by the mother, therefore, those results are subject to underreporting. Despite these limitations, our study had notable strengths; we used a clinical sample from a secluded high-risk population that is understudied and compared them with control comparisons.

## 6 Conclusion

Females in treatment for SUD and their children reported a substantial ACE burden much higher than that of controls living in the same area. Maternal ACE score was found to be positively associated with their children’s ACE score. Considering the elevated levels of ACE in this population, future research should further explore trauma experiences by mothers in SUD treatment and their children. Future studies should employ rigorous matching of cases and controls and ideally be selected from multiple treatment sites to establish generalizable results. Such research may lend additional support that the development of trauma informed interventions for the mother–child dyads is warranted.

## CRediT authorship contribution statement

**Brittany T. Smith:** Conceptualization, Methodology, Investigation, Formal analysis, Writing - original draft, Writing - review & editing. **Michael R. Brumage:** Supervision, Methodology, Writing - review & editing. **Keith J. Zullig:** Supervision, Writing - original draft, Writing - review & editing. **Elizabeth A. Claydon:** Formal analysis, Writing - review & editing. **Megan L. Smith:** Formal analysis, Writing - original draft, Writing - review & editing. **Alfgeir L. Kristjansson:** Formal analysis, Writing - original draft, Writing - review & editing.

## Declaration of Competing Interest

The authors declare that they have no known competing financial interests or personal relationships that could have appeared to influence the work reported in this paper.
